# Changes in Osteoblastic Activity in Patient Who Received Bortezomib as Second Line Treatment for Plasma Cell Myeloma: A Prospective Multicenter Study

**DOI:** 10.1155/2014/245247

**Published:** 2014-06-23

**Authors:** Ki-Seong Eom, Seok Jin Kim, Je-Jung Lee, Cheolwon Suh, Jin Seok Kim, Sung-Soo Yoon, Byung Soo Kim, Hye Jin Kang, Young Jin Choi, Chul Soo Kim, Yang Soo Kim, Jae-Yong Kwak, Yoo Jin Kim, Young Don Joo, Yeung-Chul Mun, Deog Yeon Jo, Joon Seong Park, Chi-Young Park, Sung-Hyun Kim, Chang-Ki Min

**Affiliations:** ^1^Division of Hematology, Department of Internal Medicine, Catholic Blood and Marrow Transplantation Center, Seoul St. Mary's Hospital, College of Medicine, The Catholic University of Korea, 222 Banpo-daero, Seocho-gu, Seoul 137-701, Republic of Korea; ^2^Department of Medicine, Samsung Medical Center, Sungkyunkwan University School of Medicine, Seoul 135-710, Republic of Korea; ^3^Department of Hematology-Oncology, Chonnam National University Medical School, Jeollanam-do 519-763, Republic of Korea; ^4^Department of Oncology, Asan Medical Center, University of Ulsan College of Medicine, Seoul 138-736, Republic of Korea; ^5^Department of Internal Medicine, Yonsei University College of Medicine 120-752, Republic of Korea; ^6^Department of Internal Medicine, Seoul National University College of Medicine, Seoul 110-744, Republic of Korea; ^7^Department of Medicine, Korea University Medical Center, Seoul 136-705, Republic of Korea; ^8^Department of Internal Medicine, Korea Cancer Center Hospital, Seoul 139-706, Republic of Korea; ^9^Department of Internal Medicine, Pusan National University School of Medicine, Busan 602-739, Republic of Korea; ^10^Inha University Hospital, Incheon 400-711, Republic of Korea; ^11^Departments of Hematology/Oncology and Internal Medicine, Kosin University Gospel Hospital, Busan 602-702, Republic of Korea; ^12^Department of Internal Medicine, Chonbuk National University Hospital, Jeonju 561-712, Republic of Korea; ^13^Department of Internal Medicine, Inje University College of Medicine, Busan 612-896, Republic of Korea; ^14^Department of Internal Medicine, Ewha Womans University School of Medicine, Seoul 158-710, Republic of Korea; ^15^Department of Internal Medicine, Chungnam National University College of Medicine, Daejeon 310-721, Republic of Korea; ^16^Department of Hematology-Oncology, Ajou University Hospital, Suwon 443-380, Republic of Korea; ^17^Department of Internal Medicine, Chosun University College of Medicine, Gwangju 501-717, Republic of Korea; ^18^Department of Internal Medicine, Dong-A University College of Medicine, Busan 602-715, Republic of Korea

## Abstract

We conducted a prospective multicenter study identifying the role of bortezomib in patients with relapsed or refractory plasma cell myeloma (PCM) in bone resorption and formation via bone turnover markers. A total of 104 patients received at least 1 cycle of bortezomib. Most of them had advanced disease (*n* = 89). Among them, 75 patients completed 4 cycles of treatment. Most of the patients (81.7%) were treated in combination with steroid. After the 4th cycle treatment, 47 of 75 patients achieved CR, nCR, VGPR, and PR (64.4%), while 26 patients achieved less than PR (35.6%). The proportion of patients who achieved ≥ PR increased as patients received more treatment cycles, reaching 90% after the 8th cycle. DKK-1 levels decreased significantly posttreatment. Bone formation markers (bALP and OC) and osteoclast regulator such as sRANKL also decreased significantly. These findings were observed primarily in patients who received steroid and who had a longer disease duration. While sRANKL demonstrated significant reduction posttreatment, osteoprotegerin (OPG) level did not significantly change posttreatment, resulting in a decreased sRANKL/OPG ratio (*P* = 0.037). In conclusion, our clinical data suggest that treatment with bortezomib and steroid may rearrange the metabolic balance between osteoblast and osteoclast activities in PCM.

## 1. Introduction

Plasma cell myeloma (PCM) is a neoplasm of plasma cells characterized by the appearance of monoclonal immunoglobulin, that is, bone pain caused by osteolytic lesions and pathologic fracture, hypercalcemia, renal insufficiency, and anemia [1]. Prognosis of PCM is variable, with the survival ranging from several months to over 10 years.

Myeloma bone disease is the result of increased destruction of bone that cannot be compensated by new bone formation, which develops in approximately 80% of the patients. Myeloma cells activate osteoclasts through various osteoclast activating substances and suppress the activity of osteoblasts, causing an imbalance between bone resorption and formation. This imbalance induces myeloma-related bone problems, which are the most debilitating manifestation of the disease and have direct relationship with patient's quality of life. Therefore, controlling myeloma bone disease has been regarded to be an important goal of treatment. Currently, various types of bisphosphonate have been used for myeloma bone disease [2]. Bisphosphonates inhibit osteoclastic function which reduces bone resorption and bone pain, improve patient's performance, and preserve one's quality of life [3–5]. However, bisphosphonates are known to be associated with renal impairment [2] and an increase in the risk of osteonecrosis of the jaw [6]. Furthermore, these agents have been shown to have little impact on osteoblasts.

Bortezomib (Velcade) has been approved to treat PCM as it activates osteoblasts [7–13] and suppresses osteoclasts [7, 10, 11, 13–15], in addition to an antimyeloma effect, ultimately leading to bone formation [16–18]. Preclinical studies indicate that bortezomib induces mesenchyme stem cells to preferentially undergo osteoblastic differentiation, resulting in increased bone formation and rescue from bone loss [19]. Clinically, bortezomib containing treatment for relapsed or refractory PCM demonstrated an improvement in bone lesions on radiologic examination [20] with an association of direct bone anabolism. These studies indicate that bortezomib provides a differential advantage from other agents used for treatment of PCM. It should be preferentially considered as a treatment method as bone disease has significant impact on mortality and morbidity of the patients. Until now, there have been no other antimyeloma agents to have anabolic effect on bone. In clinical practice, however, most of treatment regimens contain steroid, which exerts differential effects on bone metabolism. As far as we know, there have not been much data exploring the role of bortezomib in combination with or without steroid specifically in bone metabolism. Therefore, we conducted a prospective multicenter study to identify the role of bortezomib along with steroid among patients with relapsed or refractory PCM in bone resorption and formation using bone turnover markers prior to and after treatment.

## 2. Patients and Methods

### 2.1. Patients

Eligible patients were at least 18 years old and treated with bortezomib as a second line treatment for relapsed or refractory PCM. All patients provided a written informed consent to blood sampling to measure serum bone markers before and after therapy. Exclusion criteria included hypersensitivity to bortezomib, inadequate organ function, and pregnancy. The study was approved by each institutional review board of the participation centers in accordance with the Declaration of Helsinki.

### 2.2. Study Design and Treatment

Between March 2008 and June 2009, this multicenter prospective study was conducted at 20 centers in the Republic of Korea to investigate the role of bortezomib in bone resorption and formation. The details on treatment schedule were given elsewhere [9]. Briefly, bortezomib was administered as intravenous bolus (1.3 mg/m^2^ twice weekly in a 21-day cycle) in various combinations with other chemotherapeutic agents including steroid, thalidomide, or alkylating agents. If patients experienced grade 4 hematologic toxicities or nonhematologic toxicities ≥grade 3 other than peripheral neuropathy related to bortezomib, bortezomib was withheld until toxicity recovered to grade ≤1. Once the toxicity resolved, bortezomib was readministered with a reduced dose of 25% (1.3 mg/m^2^ to 1.0 mg/m^2^; 1.0 mg/m^2^ to 0.7 mg/m^2^).

The primary end point was changes in the levels of bone markers before and after 4 cycles of bortezomib infusion. Secondary end points included complete response (CR) rate, overall response rate, correlation between changes in bone markers and response rate, numeric rating scale (NRS) pain score, and safety profiles.

### 2.3. Assessment

Blood samples were obtained before and after bortezomib infusion at baseline, after 4 cycles, and after 5–8 cycles of treatment, respectively. Patients who received at least 4 cycles of bortezomib with blood samples from each bortezomib infusion were included for the evaluation. As an osteoclast regulator, soluble receptor activator of nuclear factor-*κ*B ligand (sRANKL) and osteoprotegerin (OPG) were measured. Dickkopf-1 (DKK-1) was measured as an osteoblast inhibitor. Bone-specific alkaline phosphatase (bALP) and osteocalcin (OC) were measured as bone formation indices.

Assessment of response, relapse, and progression was based on serum and/or urine M-protein quantification, bone marrow evaluation, and skeletal survey using European Bone and Marrow Transplantation (EBMT) criteria [21] and International Myeloma Working Group (IMWG) criteria [22]. Pain was graded using NRS (numeric rating scale), and pain scale was measured before day 1 of each bortezomib cycle. The pain was regarded as mild, moderate, and severe if the visual analogue scale (VAS) was 0 to 3, 4 to 6, and 7 to 10, respectively.

### 2.4. Statistical Analysis

Differences between pre- and post-bortezomib values of the studied parameters were evaluated using the Wilcoxon rank sum test. Differences between pretreatment and posttreatment values within each group were analyzed. Results were considered statistically significant when *P* < 0.05.

## 3. Results

A total of 104 patients received cycle 1 bortezomib treatment (Table 1). Among them, 75 patients (72.1%) completed the 4th treatment cycle and 23 patients (22.1%) finished 8 cycles of treatment. The reasons for not completing the whole treatment cycle were the termination of the clinical study as decided by the investigators (*n* = 18), adverse drug reactions or death of any cause (*n* = 16), obtaining CR before the 8th treatment cycle (*n* = 7), withdrawal by patients (*n* = 5), loss of followup (*n* = 3), and other reasons (*n* = 32). The median of treatment cycles for all patients was 4.6 ± 2.2. The majority of the patients were stages II and III (*n* = 89, 85.6%) via the international staging system. The first line of treatment included vincristine + adriamycin + dexamethasone (VAD), melphalan + prednisolone (MP), and autologous stem cell transplantation (ASCT). Most of the patients received bortezomib in combination with other agents, with the most common one being steroid (81.7%).

### 3.1. Response to Bortezomib Treatment

**Figure 1:**
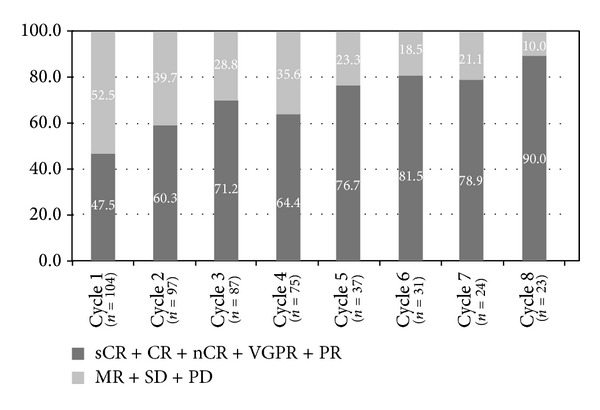
Response to bortezomib treatment.

Of 75 patients who finished the 4th cycle treatment, 64% of patients who finished the 4th cycle treatment achieved ≥PR, while the remaining 36% did not reach PR (Figure 1). Meanwhile, the proportion of patients who achieved ≥PR increased as patients received more treatment cycles, reaching 90% after the 8th cycle.

**Figure 2:**
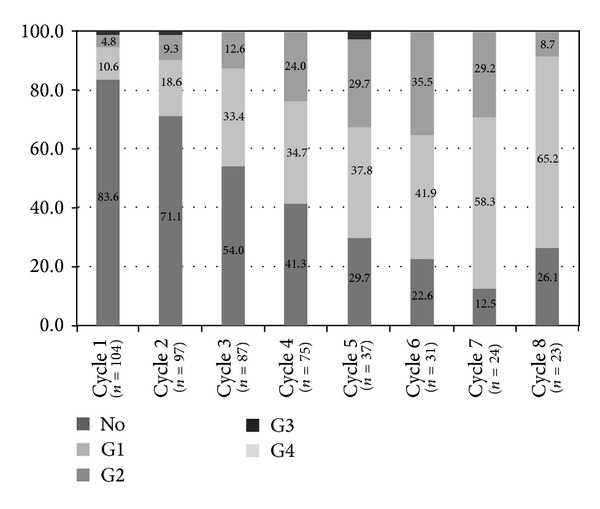
Peripheral neuropathy according to bortezomib treatment cycles.

The median VAS for pain at baseline and after the 4th cycle was 3.1 ± 2.3 and 3.1 ± 2.2, respectively. Although there was no significant reduction in bone pain in terms of total VAS after cycle 4, the proportion of patients who reported severe bone pain (VAS ≥7) decreased from 11.5% to 6.7% after cycle 4. The number of skeletal lesions did not show significant changes (2.5 ± 1.9 at baseline, 2.5 ± 1.9 after cycle 4, and 3.3 ± 2.9 after cycle 8, resp.) after bortezomib treatment. Most of the patients experienced peripheral neuropathy with the progress of treatment, but the severity of peripheral neuropathy was grade I/II in most cases (58.7% after cycle 4 and 73.9% after cycle 8, resp.; Figure 2). The proportion of grade II neuropathy decreased while that of grade I neuropathy increased as treatment progressed.

### 3.2. Changes in Bone Markers

There were no significant differences in bone turnover markers (OC, bALP, DKK-1, sRANKL, OPG, and sRANKL/OPG ratio) at baseline according to gender and age except for OPG. OPG levels were significantly higher in patients aged ≥65 years (*P* = 0.002). Changes in the level of bone markers before treatment and posttreatment are shown in Table 2. sRANKL demonstrated a significant reduction posttreatment (*P* = 0.011), and OPG levels did not change significantly, resulting in a decreased sRANKL/OPG ratio (*P* = 0.037). Despite a significant decrease in levels of DKK-1 (*P* = 0.035), an osteoblastic inhibitor, after bortezomib treatment, the levels of OC and bALP were also significantly lower posttreatment (*P* < 0.0001 and 0.004, resp.). The inconsistency in changes of DKK-1 and OC/bALP may result from the long disease duration (relapsed and/or refractory status) and advanced disease status (stages III and IV, 85.6%). The type of agents used in combination with bortezomib also could be associated with the discordance. Therefore, we analyzed the changes in OC and bALP before and after bortezomib treatment according to disease duration and combination with corticosteroid. The levels of OC and bALP decreased more significantly in longer diagnosis-treatment period group (≥6 months, longer prevalence period group) than those in the shorter group (Table 3). Influences of steroid in combination with bortezomib on bone markers are also shown in Table 3. Serum OC and bALP levels decreased significantly in the steroid combination group (*P* = 0.0002 and 0.001, resp.), while the same bone marker changes were not significant in the bortezomib single-treatment group.

## 4. Discussion

There have been multiple studies of bortezomib, alone and in combination, in both previously untreated and relapsed/refractory PCM patients and its effect on bone remodeling markers [8, 10, 12, 13, 18, 23–26]. Most studies revealed that biomarkers of osteoblastic activity increased and the levels of osteoblast inhibitors decreased, while the levels of markers of osteoclastic activity have also been shown to decrease. Bortezomib treatment is associated with increased ALP and OC, while depressing sRANKL and OPG. Additionally, bortezomib was associated with a decrease in DKK-1 levels.

In our study, despite bortezomib treatment, the levels of biomarkers related to bone formation (bALP and OC) substantially decreased, especially in the patients with longer prevalence period and steroid combination. Most of the patients (81.7%) in our study were treated in combination with corticosteroid. Overall patterns in changes of bone markers (decreased levels in OC and bALP) were similar to those in steroid combination group, but these patterns were not observed in bortezomib single-treatment group and the shorter prevalence group. In general, the effects of steroid are represented by a reduction in bone formation markers and a trend towards an increase or no change in bone resorption markers, which implies that steroids have a dominant effect on bone formation rather than bone resorption [27]. As a result, corticosteroid has been known to depress serum OC level, mainly due to the reduction in the rate of bone formation [28]. bALP, an enzyme released from osteoblasts, decreased in patients receiving steroid due to the reduction in the number of osteoblasts. Therefore, the reduction seen in the level of bone formation-related markers, bALP and OC in our study, is thought to result from the dominant effect of corticosteroid over bortezomib on bone formation. In a similar context, patients with longer prevalence duration also have a probability of longer exposure to steroid at a higher dose, which could explain the similar pattern in biomarkers in that group. The fact that significantly low levels of OC and bALP were found in the longer prevalence group before bortezomib therapy could also explain the decreased level posttreatment in the group. Terpos et al. also showed that the combination of bortezomib with melphalan, dexamethasone, and intermittent thalidomide (VMDT) did not increase the bone formation markers (bALP and OC) although a reduction in DKK-1 levels was observed [12]. On the other hand, bortezomib was associated with a decrease in DKK-1 levels. Consistent with these results, our study showed decreased serum DKK-1 level posttreatment. However, patients in steroid combination group did not show statistical significance in DKK-1 level (data not shown). Decreased production of DKK-1 by bortezomib might be offset by increased production of DKK-1 by steroid. Generally, concentrations of osteoclast regulators such as sRANKL and OPG were shown to be reduced following treatment with bortezomib in previous reports [12]. In our study, sRANKL level diminished significantly, whereas there were no significant changes in OPG levels posttreatment, resulting in marked reduction in sRANKL/OPG ratio. The reasons for the inconsistency of results in our study remain unclear but may be explained by the predominant effect of steroid on bone formation rather than increased bone resorption. As for bone formation markers, such as OC and bALP, they appear to decrease significantly due to the predominant effect of steroid on bone formation, while the effect of bortezomib predominates for osteoclast regulators such as sRANKL.

As the treatment in our study also included combination with dexamethasone in most cases and some patients received thalidomide, it is difficult to interpret exactly the role of bortezomib in the bone markers. The effect of corticosteroid on biochemical markers of skeletal turnover can be varied according to a number of biases. These include the different effects on different markers; different steroids, exposure time, and amount of administered steroids; different routes of administration, evaluation of the effect on normal subjects (men versus women, fertile versus postmenopausal subjects, or young versus adults) or on patients with disease; or the inability to control parameters (gonadal function, parathyroid function, vitamin D status, etc.) that* per se* influence turnover [27].

Overall, these clinical data suggest that the combination treatment with bortezomib and steroid could rearrange the metabolic balance between osteoblast and osteoclast activities in PCM and the effects of corticosteroid predominate in inhibiting bone formation. Bortezomib appears to dominate in inhibiting bone resorption, while the effect of steroid is minimal in the inhibition of bone resorption.

## Figures and Tables

**Table 1:** Patient characteristics.

Total enrolled patients, *n* (%)	104
Cycle 1	104 (100.0)
Cycle 4	75 (72.1)
Cycles of bortezomib treatment, mean ± SD	4.6 ± 2.2
Sex, *n*, M/F (%)	56/48 (53.9/46.1)
Age at treatment, *n*, <65/≥65 (%)	67/37 (64.4/35.6)
ECOG performance status, *n* (%)	
0	23 (22.1)
1	56 (53.9)
2	23 (22.1)
3	2 (1.9)
4	0
Quantity of M-protein (g/L) in SPEP, median (range)	1.3 (0.0–6.7)
Hb ≤ 10.0 g/dL, *n*/median (%/range)	41/10.7 (39.4/6.9–15.3)
Platelet, median, *n* (range)	203.0 (30.0–760.0)
Creatinine, median (range)	0.98 (0.5–5.6)
Calcium (g/dL), median (range)	8.7 (6.5–11.1)
LDH > 472 IU/L, *n*/median (%/range)	20/350.0 (19.2/15.0–1452.0)
Albumin (g/L), median (range)	3.7 (2.4–6.4)
Stage, *n* (%) Durie-Salmon Staging System	
I	13 (12.5)
II	21 (20.2)
III	70 (67.3)
Stage, *n* (%) International Staging System	
I	15 (14.4)
II	44 (42.3)
III	45 (43.3)
Previous treatment, *n* (%)	
VAD	37 (35.6)
MP	30 (28.9)
Dexamethasone	29 (27.9)
ASCT	21 (20.2)
Thalidomide	19 (18.3)
Others	21 (20.2)

VAD: vincristine + Adriamycin + dexamethasone; MP: melphalan + prednisolone; ASCT: autologous stem cell transplantation.

**Table 2:** Changes in bone markers.

Variables	Pretreatment	Posttreatment	Difference^†^	*P*
*n* (%)	75 (100.0)	73 (97.3)	73 (97.3)	
Osteocalcin ECLIA (ng/mL)	17.7 ± 18.6	13.7 ± 16.0	4.0 ± 15.9	**<0.0001**
Bone ALP (U/L)	25.9 ± 16.2	21.8 ± 11.2	4.2 ± 14.3	**0.004 **
DKK-1 (pmol/L)	136.4 ± 86.3	116.3 ± 58.8	20.2 ± 78.6	**0.035 **
sRANKL (total, pmol/L)	75.1 ± 86.8	48.4 ± 47.2	28.1 ± 101.6	**0.011 **
OPG (pmol/L)	3.9 ± 1.8	4.0 ± 1.9	−0.1 ± 1.1	0.464
sRANKL/OPG ratio	22.2 ± 34.4	13.5 ± 16.1	9.4 ± 36.6	**0.037 **

ECLIA: electrochemiluminescence immunoassay.

^†^Pretreatment value − posttreatment value.

**Table 3:** Changes in bone markers according to the duration of the disease and steroid combination.

	Osteocalcin	P*	Bone ALP	P**
Disease duration		0.009		0.050
<6 months				
Pretreatment	18.6 ± 13.9		29.3 ± 18.8	
Posttreatment	19.6 ± 24.3		25.7 ± 13.0	
Difference	1.2 ± 24.1		4.2 ± 17.0	
P*	0.058		0.088	
≥6 months				
Pretreatment	13.9 ± 19.6		23.4 ± 14.0	
Posttreatment	10.1 ± 9.7		18.0 ± 6.4	
Difference^†^	4.9 ± 13.3		5.5 ± 12.4	
P*	**<0.0001**		**0.002**	

Steroid				
Combination				
Pretreatment	16.5 ± 18.0		27.3 ± 18.0	
Posttreatment	13.6 ± 15.6		22.0 ± 10.5	
Difference	4.2 ± 18.7		5.4 ± 15.7	
P*	**0.0002**		**0.001**	
Without combination				
Pretreatment	15.5 ± 12.1		22.7 ± 9.3	
Posttreatment	20.5 ± 30.9		20.9 ± 13.2	
Difference	−2.8 ± 22.3		2.1 ± 9.0	
P*	0.153		0.275	

*P value represents difference from pretreatment value to posttreatment value within each group.

**P value represents difference of treatment outcome by prevalence duration.

^†^Posttreatment value − pretreatment value.
